# Theory of Nervous Force

**Published:** 1872-04

**Authors:** 


### ARTICLE VIII.

### *Theory of Nervous Force.*

In the *Medical and Surgical Reporter* are published the conclusions of Dr. Charles B. Radcliffe:

"Instead of regarding the state of action in nerve and muscle as a manifestation of vitality, there is, indeed, reason to believe that it must be brought under the dominion of physical law in order to be intelligible, and that a different meaning, also based upon pure physics, must be attached to the state of rest.

There is reason to believe that all kinds of electricity act on nerve and muscle by way of charge and discharge, the charge antagonizing, the discharge permitting the state of action.

There is reason to believe that the blood acts upon nerve and muscle, not by causing the state of action, but by antagonizing it.

There is reason to believe that nervous influences act upon nerve and muscle, not by causing the state of action, but by antagonizing it.

The whole case is simple enough. It would seem indeed, 1. That the sheaths of the fibres in nerve and muscle are capable of being charged like Leyden jars, and that during the state of rest they are so charged. 2. That the sheaths of the fibres are highly elastic. 3. That the fibres of muscles are elongated during the state of rest, by the charge with which their sheaths are charged, the mutual attraction of the two opposite electricities disposed Leyden-jar-wise upon two surfaces of the sheaths, compressing the elastic substance of the sheaths, and so causing elongation of the fibre in proportion to the amount of the charge. 4. That the muscular fibres contract when the state of rest changes for that of action, because the charge which causes the state of elongation during rest is then discharged, and because this discharge leaves the fibres free to return by virtue of their elasticity, simply from the state of elongation in which they had been previously kept by the charge. The degree of contraction is proportional to the degree of elongation previously existing.

That the fibres of nerve are not affected in the same way as the fibres of muscle by the charge and discharge of electricity, because the sheaths of the fibres may be wanting in the requisite degree of elasticity. 5. That the blood antagonizes the state of action in nerve and muscle by helping to keep up the natural electrical charge which antagonizes action. 6. That nervous influences antagonize the state of action in nerve and muscle, by helping to keep up the natural electric charge which antagonizes action. 7. That diminished efflux of blood to certain nerve centres leads to successive action in nerve and muscle, by disturbing the electric equilibrium of the nervous system, which is maintained during the state of rest, this disturbance causing a partial reversal in the relative position of the two electricities with which the sheaths of the fibres are charged, and so necessitating the discharge which is the basis of action."

---

